# CTX-M variants on the move: Tracking resistance in *Escherichia coli* and *Klebsiella* spp. across humans, livestock and wildlife in Western Uganda

**DOI:** 10.1016/j.onehlt.2026.101407

**Published:** 2026-04-07

**Authors:** Judith Guitart-Matas, Andrea Dias-Alves, Ignasi Marco, Laura Carrera-Faja, Jesús Muro, Josephine Tushabe, Edrine Kayaga, Dickson Ndoboli, Fred Babweteera, Carol Asiimwe, Oscar Cabezón, Lourdes Migura-Garcia

**Affiliations:** a IRTA. Programa de Sanitat Animal, CReSA. Campus de la UAB, 08193 Bellaterra, Catalonia, Spain; b Laboratori de Recerca en Microbiologia i Malalties Infeccioses, Institut d'Investigació i Innovació Parc Taulí (I3PT), Hospital Universitari Parc Taulí, Universitat Autònoma de Barcelona, 08208 Sabadell, Catalonia, Spain; c Wildlife Conservation Medicine Research Group (WildCoM), Departament de Medicina i Cirurgia Animals, Universitat Autònoma de Barcelona, 08193 Bellaterra, Catalonia, Spain; d Daktari, Urb. La Solana 35, AD700, Escaldes, Andorra; e Central Diagnostic Laboratory, COVAB, Makerere University, P.O. Box 7062, Kampala, Uganda; f Budongo Conservation Field Station, Masindi, Uganda; g Unitat mixta d'Investigació IRTA-UAB, Centre de Recerca en Sanitat Animal (CReSA), Campus de la Universitat Autònoma de Barcelona (UAB), 08193 Bellaterra, Catalonia, Spain

**Keywords:** Antimicrobial resistance, *Escherichia coli*, *Klebsiella* spp., Livestock, Uganda, Wildlife

## Abstract

Antimicrobial resistance (AMR) is a major global public health challenge. The AMR burden is disproportionately higher in low- and middle-income countries (LMICs), where widespread and inappropriate antimicrobial use promotes the emergence and spread of resistance. The expanding human population and increasing habitat fragmentation of natural areas force wildlife into greater contact with humans and livestock, facilitating AMR transmission. This study aimed to assess the occurrence of extended spectrum cephalosporin (ESC)-resistant Enterobacterales as an indicator for AMR burden across the human-animal-environment interface in rural areas from Western Uganda. Samples were collected from humans (*n* = 65), livestock (*n* = 137), wildlife (*n* = 301), and environmental sources (*n* = 52) in rural areas with varying levels of human-animal interaction. ESC-resistant *Escherichia coli* (*n* = 58) and *Klebsiella* spp.(n = 5) were identified and antimicrobial susceptibility testing was performed. Thereafter, whole genome sequencing was carried out in ESC-resistant isolates (*n* = 63) to characterize resistance genes and lineages, and further compared them with previously published sequences from Uganda. A high prevalence of the CTX-M-15 gene was observed, alongside CTX-M-27 and OXA-1. Multiple sequence types (STs) were detected for both *E. coli* and *Klebsiella* spp., though globally dominant extraintestinal pathogenic *E. coli* (ExPEC) lineages were infrequent. The wide occurrence of ESC-resistant bacteria in community settings and wildlife underlines the interconnectedness of AMR transmission. These findings emphasize the need for a comprehensive One Health approach to better understand AMR transmission dynamics in rural areas from Western Uganda.

## 1 Introduction

Antimicrobial resistance (AMR) is a major global public health challenge, threatening both, human and animal health [1], [2], [3]. In 2019, bacterial AMR was estimated to have directly caused over one million deaths worldwide and contributed to several million additional deaths, highlighting its substantial global impact [4]. The burden is disproportionately higher in low- and middle-income countries (LMICs), where widespread and often inappropriate antimicrobial use contributes to the emergence and spread of resistance [5].

The Enterobacterales is an order of Gram-negative bacteria that naturally inhabit the gastrointestinal tract of warm-blooded animals, but they also cause a wide range of diseases, including urinary tract infections (UTIs), gastroenteritis and septicaemia [6]. Beta-lactams, including penicillins, cephalosporins, monobactams and carbapenems, are the most widely used antimicrobial agents worldwide to treat infections caused by these pathogens. Although overall consumption remains higher in high-income countries (HICs), antibiotic consumption rates are increasing in LMICs and are expected to surpass those in HICs due to the greater burden of infectious diseases [7]. Since the early 2000s, there has been an increased trend of extended-spectrum cephalosporin (ESC)-resistant Enterobacterales, especially *Escherichia coli* and *Klebsiella pneumoniae*, resistant to third-generation cephalosporins, mainly due to the global spread of extended-spectrum beta-lactamases (ESBLs) and plasmid-mediated AmpC beta-lactamases [8], [9]. Furthermore, these ESBL-producing organisms are often multi-drug resistant (MDR), leading to higher mortality rates, longer hospital stays, and increased costs compared to antibiotic-susceptible strains [10]. CTX-M is the most disseminated ESBL type and has been widely reported in humans, domestic and wild animals, and environmental sources worldwide [11], [12], [13], [14].

The expanding human population and increasing habitat fragmentation of natural areas force wildlife into greater contact with humans and livestock [15], [16]. This increased interaction substantially contributes to the spread of pathogens [17]. Despite the substantial progress made in the study of the epidemiology of multi-host infections, the role of wildlife reservoirs in the dissemination of AMR in the natural environment remains poorly understood [18], [19]. Wildlife often comes into contact with contaminated water sources and soil, which can harbour resistant bacteria from agricultural runoff and human waste [20]. Therefore, monitoring the burden of AMR bacteria in wildlife serves as a valuable tool to assess human impacts on the environment, and provides insights into the pathways of resistance transmission, enabling the development of more effective strategies to mitigate the spread of AMR.

The aim of this study was to determine the occurrence of ESC-resistant Enterobacterales as an indicator of the burden of resistance across the human-animal-environmental interface in Uganda.

## 2 Materials and methods

### 2.1 Study area

**Fig. 1:**
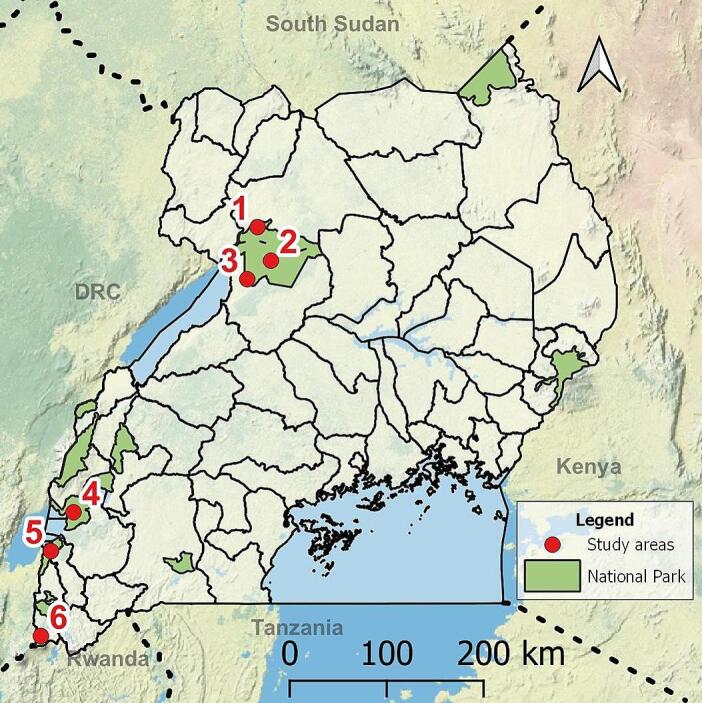
Map of Uganda and the areas of study: Murchison Falls NP-Northern sector (1), Murchison Falls NP-Southern sector (2), Budongo Forest (3), Queen Elizabeth NP-Northern sector (4), Queen Elizabeth NP-Southern sector (5) and Mgahinga Gorilla NP (6).

This study was conducted from 2017 to 2019 in six areas of Western Uganda (Fig. 1): Murchison Falls National Park-Northern sector (MFNP-N; 2°18′28″N, 31°33′40″E), Murchison Falls National Park-Southern sector (MFNP-S; 2°11′15″N, 31°46′53″E), Budongo Central Forest Reserve (BCFR; 1°43′27″N, 31°32′45″E), Queen Elizabeth National Park-Northern sector (QENP-N; 0°8′14″S, 30°02′28″E), Queen Elizabeth National Park-Southern sector (QENP-S; 0°33′00”S, 29°53′00″E), and Mgahinga Gorilla National Park (MGNP; 1°22′10″S, 29°38′25″E).

These areas are situated within the Albertine Rift and exhibit different degrees of interaction between livestock, wildlife, and humans. Briefly, MFNP-N and QENP-S are inhabited by wildlife, without any livestock presence. Then, interspecies contact is rare. In contrast, in MFNP-S and QENP-N, domestic animals and human settlements are present, facilitating indirect interactions with wildlife. Similarly, in BCFR, contact between chimpanzees, baboons, domestic animals and humans is common, as these primates forage outside the forest from croplands. Although a stone wall separates MGNP from inhabited areas, this wall is permeable for wildlife, facilitating indirect interactions with other species. Nevertheless, all these areas are constantly frequented by locals and tourists working or visiting the National Park (NP).

### 2.2 Sample collection

Overall, 52 environmental (water) and 503 faecal samples from livestock (*n* = 137), wildlife (*n* = 301) and humans (*n* = 65) were collected (Table 1). Human and livestock samples were collected from areas surrounding the National Parks previously mentioned. Faecal and water samples were collected aseptically using sterile plastic sample bags and BD Falcon tubes, respectively, and maintained at 4–8 °C until laboratory analyses (<72 h). All field and laboratorial procedures were approved by the Uganda Wildlife Authority (UWA) (EDO/35/01 and COD/96/02), the Higher Degrees Research Committee of the College of Veterinary Medicine, Animal Resources and Biosecurity at Makerere University (SBLS/HDRC/20/011), and the School of Biomedical Sciences Research and Ethics Committee (SBS-REC-824).

**Table 1:** Total number of samples analysed, number and percentage of samples where *E. coli* and *Klebsiella* spp. were detected, and number of isolates resistant to ceftazidime (CAZ-R).

	N samples	*E. coli* detection (%)	*Klebsiella* spp. detection (%)	CAZ-R
Environment	52	17 (32.7)	0	0
Water	52	17 (32.7)	0	0
Livestock	137	122 (89.1)	0	14
Cattle	57	51 (89.5)	0	6
Goat	58	49 (84.5)	0	8
Sheep	22	22 (100)	0	0
Wildlife	301	288 (95.7)	0	10
Baboon *(Papio anubis)*	43	41 (95.3)	0	8
Blue monkey *(Cercophitecus mitis)*	2	2 (100)	0	0
Buffalo *(Syncerus caffer)*	61	55 (90.2)	0	0
Bushback *(Tragelaphus scriptus)*	2	2 (100)	0	0
Chimpanzee *(Pan troglodytes)*	108	108 (100)	0	1
Duika *(Sylvicapra grimmia)*	2	2 (100)	0	0
Elephant *(Loxodonta africana)*	37	32 (86.5)	0	0
Giraffe *(Giraffa camelopardalis)*	17	17 (100)	0	0
Ugandan kob *(Kobus kob thomasi)*	29	29 (100)	0	1
Human	65	57 (87.7)	5 (7.7)	39
TOTAL	555	484 (87.2)	5 (0.9)	63

### 2.3 Bacteria isolation, identification and antimicrobial susceptibility testing

Faecal and water samples were collected for microbiological analysis. Human faecal samples were self-collected using sterile containers, while animal faecal samples were collected directly from the ground immediately after defecation using sterile tools to avoid environmental contamination. All samples were transported to the laboratory under refrigerated conditions and processed within 24 h. Water samples were collected from shared water sources (e.g., drinking troughs and natural ponds) used by both wildlife in national parks and livestock, using sterile bottles. Samples were transported under cooled conditions and processed immediately upon arrival to the laboratory. For water samples, 100 mL were filtered through a 0.22 μm polycarbonate membrane filter (ISOPORE 0.2 μm GTTP 47 mm diameter, Millipore) after homogenisation by agitation, following the Tricycle protocol [21]. The membrane was then resuspended in phosphate-buffered saline (PBS) prior to further processing. Faecal samples and resuspended water filtrates were inoculated onto both MacConkey agar (Oxoid, Basingstoke, United Kingdom) and MacConkey agar supplemented with 1 mg/L of ceftriaxone and incubated at 37 °C for 24 h. One colony per positive sample morphologically compatible with *E. coli* or *Klebsiella* spp. was again subcultured on the same isolation agar. The inoculated agar plates were then incubated at 37 °C for 24 h to obtain pure colonies. The colonies were biochemically identified, and isolates were stored at −80 °C in brain heart infusion with 20% glycerol for further studies.

The Kirby-Bauer disk diffusion test (DDT) method was used to detect antibiotic susceptibility [22]. Six antibiotic disks were tested: ampicillin (10 μg), ceftazidime (30 μg), chloramphenicol (30 μg), gentamicin (10 μg), sulphonamide (240 μg) and tetracycline (30 μg). Minimal inhibitory concentration (MIC) was determined by microbroth dilution method (Sensititre®, ThermoFisher Scientific, Spain) following guidelines of the European Committee on Antimicrobial Susceptibility Testing (EUCAST), in those isolates harbouring resistance to third-generation cephalosporins obtained in the supplemented agar and confirmed by the Kirby-Bauer DDT method. Antimicrobials and concentrations tested were as follows: amikacin (4 to 128 μg/mL), ampicillin (1 to 32 μg/mL), azithromycin (2 to 64 μg/mL), cefotaxime (0.25 to 4 μg/mL), ceftazidime (0.25 to 8 μg/mL), ciprofloxacin (0.015 to 8 μg/mL), chloramphenicol (8 to 64 μg/mL), colistin (1 to 16 μg/mL), gentamicin (0.5 to 16 μg/mL), meropenem (0.03 to 16 μg/mL), nalidixic acid (4 to 64 μg/mL), sulfamethoxazole (8 to 512 μg/mL), tetracycline (2 to 32 μg/mL), tigecycline (0.25 to 8 μg/mL) and trimethoprim (0.25 to 16 μg/L). Isolates were classified as wild type (susceptible) or non-wild type (resistant), based on epidemiological cut-off values (ECOFF) defined by EUCAST (https://www.eucast.org/).

### 2.4 Whole genome sequencing (WGS), in silico serotyping and identification of resistance profiles

Nucleic acid extraction of all ESC-resistant *E. coli* and *Klebsiella* spp. was performed using the DNeasy Ultraclean Microbial Kit (Qiagen) following the manufacturer's instructions. Quality and concentration of the DNA were determined with the NanoDrop 2000 Spectrophotometer and a Qubit dsDNA BR Assay Kit (Fisher Scientific). Genomes were sequenced with the Illumina NovaSeq platform (Illumina Inc., San Diego, CA) at the Earlham Institute (2 × 250 bp paired-end chemistry). Sequencing reads were trimmed using Trimmomatic v0.39 with a four-base sliding window to cut when the average Phred quality scores dropped below 15 and reads shorter than 36 bp were excluded [23]. Draft genomes were generated de novo using the SPAdes v3.14.1 assembler performing a preliminary read error correction based on Hamming graphs and Bayesian subclustering and including the BWA mismatch corrector tool [24]. QUAST v5.2.0 software was used to assess the quality of final assemblies [25]. Resistance profiles were identified with Resfinder v4.3.0 using ResFinder and PointFinder databases for acquired genes and point mutations, respectively [26], [27]. Predicted location of beta-lactam resistance genes was determined from assemblies using VRprofile2 [28].

Multilocus sequence typing (MLST) was determined in silico from assembled scaffolds against the PubMLST typing scheme of *E. coli* and *Klebsiella* with the mlst v2.23.0 software [29], [30]. For ESC-resistant *E. coli* sequenced genomes, phylotypes were also analysed in silico using the Clermont PCR method of the ClermonTyping software [31].

### 2.5 Phylogenomic analyses

To explore the phylogenomic relationship between the ESC-resistant *E. coli* of this study and the assemblies of Uganda origin available at the Enterobase (https://enterobase.warwick.ac.uk/; last access 31/05/2024), phylogenetic analyses were performed by core genome alignments based on single nucleotide polymorphisms (SNPs) and insertions/deletions with the Snippy v4.6.0 software [32]. A total of 124 assemblies with known source were downloaded: 108 from livestock, 14 from wildlife, and 2 from humans. The complete genome of *E. coli* strain K-12 substrain MG1655 (RefSeq Accession Number NC_000913.3) was used as reference. Gubbins v3.3.0 was implemented to analyse recombination in resultant alignments and phylogenomic trees were generated with IQ-TREE v2.2.2.3 with 10,000 bootstrap replicates and ascertainment bias correction [33], [34]. The best-fit substitution model to build the tree was the transversion model with unequal base frequencies (TVM + F + ASC + R2).

The relationship among the five *Klebsiella* spp. isolates, representing two different species, was assessed alongside genome assemblies of *K. pneumoniae* (*n* = 67) and *K. quasipneumoniae* (*n* = 9) publicly available at NCBI database. All genomes originated from Uganda (last access 23/09/2025) and belonged to different hospital patients across the country. For *Klebsiella* spp. phylogenomic profiling was performed with the Phylophlan v.3.1.68 software, mapping against the phylophlan database with DIAMOND v2.1.13.167 and performing multiple-sequence alignment with MAFFT v7.526 [35], [36], [37] Phylogenetic inference was performed with astral v1.23.4.6 and phylogeny was refined with RAxML v8.2.12 [38], [39] Final phylogenetic trees were visualized and edited with the iTOL v6.9.1 online tool [40].

### 2.6 Statistical analysis

To evaluate the differences in detection of ESC-resistant *E. coli* across different study areas, Pearson's Chi-squared test (χ2) was used, with Yates' correction when appropriate. *P*-values less than 0.05 were interpreted as statistically significant. Statistical analyses were conducted using the R version 4.3.0 [41].

## 3 Results

### 3.1 Antimicrobial susceptibility testing and resistance genes

Out of 555 samples collected, 484 (87.1%) and 5 (0.9%) isolates were confirmed to be *E. coli* and *Klebsiella* spp., respectively, each representing one individual per isolate, with varying proportions observed among the different study subjects (Table 1). Of them, 63 were selected in MacConkey supplemented with ceftriaxone, 58 *E. coli* and 5 *Klebsiella* spp., respectively. For *Klebsiella* spp. isolates, all collected from human samples, two different species were identified; three *K. pneumoniae* and two *K. quasipneumoniae.* For *E. coli* isolates, antimicrobial resistance to ampicillin, sulfamethoxazole, tetracycline, ceftazidime and chloramphenicol was detected by Kirby-Bauer DDT method in livestock, wildlife and humans. However, AMR was not observed in environmental samples (Supplementary Table S1). The highest frequency of resistance was observed for sulfamethoxazole (24%), followed by ampicillin (21.9%), tetracycline (13.8%) and ceftazidime (12%). In all cases, resistance was higher in humans, followed by livestock and wildlife. All *Klebsiella* spp. isolates were resistant to sulfamethoxazole, and ceftazidime. Isolates resistant to ceftriaxone and confirmed to exhibit resistance to ceftazidime (Table 1) were further tested for several antibiotics using MICs. According to the study areas, MGNP exhibited significantly higher levels of ESC-resistant *E. coli* compared to BFCR and MFNP (*p* = 0.004). When comparing areas with frequent interaction (QENP-N, MGNP, MFNP-S and BCFR) to those with less interaction (QENP-S and MFNP-N), ESC-resistant *E. coli* isolates were significantly more prevalent in areas with higher interaction (*p* = 0.002). However, when assessing the levels of ESC-resistant *E. coli* isolates among study subjects across different areas, no statistically significant differences were observed.

All the ESC-resistant *E. coli* and *Klebsiella* spp. isolates were resistant to ampicillin, sulfamethoxazole, cefotaxime, ceftazidime and trimethoprim, except for one *E. coli* human isolate that was susceptible to the latter and one wildlife isolate susceptible to ceftazidime (Supplementary Table S2). Ciprofloxacin, tetracycline and nalidixic acid resistance were detected in isolates from all sources, with frequencies ranging from 80 to 100%, 35.7 to 80% and 20 to 41.1%, respectively. Resistance to gentamicin and amikacin was only present in human isolates, while all individuals were susceptible to meropenem and colistin. MDR, defined as resistance to three or more classes of antibiotics [42], was present in all isolates. The number of resistances ranged from 5 to 11.

WGS detected 40 AMR genes associated with resistance to 9 different families of antibiotics, with a mean of 9.29 (SD = 3.39) resistance genes per sample in *E. coli* isolates (Supplementary Table S3). For *Klebsiella* spp., 28 AMR genes associated with 8 families of antibiotics were identified, with a mean of 13 (SD = 2) genes per isolate (Supplementary Table S4).

In the 58 *E. coli* isolates, the presence of ESC resistance genes was confirmed in all isolates except one, which was differently distributed across Western Uganda (https://microreact.org/project/qCA5iHmAHx7riG5GH2m77e-escherichiacoliuganda). The most prevalent beta-lactam resistance genes were CTX-M-15 (*n* = 53, 91.4%), OXA-1 (*n* = 6, 10.3%), SHV-12 (n = 5, 8.6%), CTX-M-27 (*n* = 3, 5.2%) and SHV-187 (*n* = 1, 1.7%). Additionally, TEM-1B (*n* = 31, 53.4%), TEM-104 (n = 1, 1.7%), and TEM-1C (*n* = 1, 1.7%) were also detected. OXA-1 was identified in 6 isolates, all in combination with CTX-M-15, one with SHV-12 and one with SHV-187. Of those isolates, 5 belonged to humans and one to a chimpanzee. Regarding aminoglycoside resistance genes, 49 isolates harboured *aph*(6)-Id (84.5%), 37 *aph*(3″)-Ib (63.8%), 12 *ant*(3″)-Ia (20.7%), 8 *aadA2* (13.8%), 6 *aadA5* (10.3%), 5 *aac*(6’)Ib-cr (8.6%), 3 harboured *aac*(3)-IIa (5.2%), one *aac*(3)-IId (1.7%) and one *ant*(2″)-Ia (1.7%). For macrolides, *mph*(A) (*n* = 13, 22.4%), and *erm*(B) (*n* = 3, 5.2%) were detected. Fluoroquinolone resistance genes were identified, specifically *qnrS1* (*n* = 41, 70.7%) and *qnrB19* (*n* = 1, 1.7%). For phenicols, *catA1* (*n* = 7, 12.1%) was the most frequent, while *fosA* conferring resistance to fosfomycin was found in two human isolates. Resistance to tetracycline was conferred by *tet*(A) (*n* = 31, 53.4%) and *tet*(B) (*n* = 10, 17.2%). For trimethoprim, the most common genes encountered were *dfrA14* (*n* = 33, 56.9%), *dfrA1* (*n* = 9, 15.5%), *dfrA12* (n = 9, 15.5%), *drfA8* (*n* = 5, 8.6%), *dfrA7* (*n* = 3, 5.2%), *dfrA5* (n = 1, 1.7%), *dfrA15* (n = 1, 1.7%), *dfrA17* (*n* = 6, 10.3%), and *dfrA19* (n = 1, 1.7%), while in sulphonamides were *sul2* (*n* = 54, 93.1%), *sul1* (*n* = 18, 31%) and *sul3* (*n* = 2, 3.4%). Analyses of point mutations within the quinolone resistance-determining regions (QRDRs), responsible for conferring resistance to this antibiotic class, detected mutations in the *gyrA*, *parC* and *parE* genes in 23 isolates. Eleven distinct combinations of mutations were identified (Table 2).

**Table 2:** Mutations combination within the quinolone resistance-determining regions (QRDRs) conferring resistance to fluoroquinolones in ESC-resistant *E. coli,* along with the origins of the samples exhibiting these mutations.

Genotype point mutations				
*gyrA*	*parC*	*parE*	N (%)	Study subject
p.S83A	–	–	1 (4.3)	Human
p.S83L	–	–	3 (13)	Human (2) and livestock (1)
p.S83L, p.D87N	p.S80I	–	2 (8.7)	Human
p.S83L, p.D87N	p.S80I	p.L416F	3 (13)	Livestock
p.S83L, p.D87N	p.S80I	p.S458A	5 (21.7)	Human (4) and wildlife (1)
p.S83L, p.D87N	p.S80I, p.E84G	–	1 (4.3)	Human
p.S83L, p.D87N	p.S80I, p.E84V	p.I529L	2 (8.7)	Human
p.S83L, p.D87N	p.S80I, p.S57T	p.S458A	1 (4.3)	Human
p.S83V	–	–	2 (8.7)	Human (1) and livestock (1)
–	–	p.I355T	2 (8.7)	Human
–	–	p.L416F, p.E460D	1 (4.3)	Human

For *Klebsiella* spp., ESC genes were detected in all isolates. The most prevalent beta-lactam resistance genes were CTX-M-15 (*n* = 4, 80%) and TEM-1B (*n* = 3, 60%). OXA-1 was found in one isolate, in combination with CTX-M-15 and SHV-145. Regarding aminoglycoside resistance genes, all isolates harboured *aph*(6)-Id and *aph*(3″)-Ib. For fluoroquinolones, *qnrS1* was detected in 3 isolates (60%). Resistance to phenicol was conferred by *catA2* (*n* = 2, 40%) and *catA1* (*n* = 1, 20%), while *fosA* was the most frequent gene conferring resistance to fosfomycin (n = 3, 60%). For tetracyclines, *tet*(A) (n = 3, 60%) and *tet*(D) (*n* = 1, 20%) were detected. For trimethoprim, the most common gene encountered was *dfrA14* (n = 4, 80%), while for sulphonamides was *sul2* (*n* = 5, 100%). As for *E. coli* isolates, point mutations conferring resistance were also analysed. Point mutations in the *ompK36*, *ompK37* and *acrR* genes were detected in all *Klebsiella* spp. isolates, with four combinations of point mutations (Table 3).

**Table 3:** Point mutations combination in ESC-resistant *Klebsiella* spp.

*ompK36*	*acrR*	*ompK37*	N (%)
p.N49S, p.L59V, p.N49S, p.L191S, p.F207W, p.A217S, p.N218H, p.D224E, p.Q227S, p.L228V, p.E232R, p.T254S, p.N304E	p.P161R, p.G164A, p.P161R, p.R173G, p.L195V, p.F197I, p.K201M	p.I70M, p.I128M	1 (20)
p.N49S, p.L59V, p.T184P	p.P161R, p.G164A, p.F172S, p.R173G, p.L195V, p.F197I, p.K201M	p.I70M, p.I128M	1 (20)
p.N49S, p.L59V, p.L191Q, p.A217S, p.N218H, p.Q227S, p.L229V, p.N304E	p.P161R, p.G164A - Frameshift, p.F172S, p.R173G, p.L195V, p.F197I, p.K201M	p.I70M, p.I128M	2⁎ (40)
p.N49S, p.L59V, p.A190W, p.L191S, p.F207W, p.A217S, p.N218H, p.D224E, p.Q227S, p.L228V, p.E232R, p.T254S, p.N304E	p.P161R, p.G164A, p.F172S, p.R173G, p.L195V, p.F197I, p.K201M	p.I70M, p.I128M	1 (20)

⁎ These mutations were detected in the two *K. quasipneumoniae* isolates.

Most genes associated with resistance to ESC in both *E. coli* and *Klebsiella* spp. were predicted to be located on plasmids (Supplementary Tables S5 and S6). For *E. coli*, of the 101 genes detected conferring resistance to beta-lactams, 49 were predicted to be in plasmids (48.5%), 10 were located in the chromosome (9.9%), and 5 were predicted to be yielded in prophage-related regions (5%). Additionally, 11 genes had an uncertain location (10.9%), and 26 were not found (25.7%). In the case of *Klebsiella* spp., of the 14 genes detected, 7 were located on plasmids (50%), 1 had an uncertain location (7.1%), and 6 were not found (42.9%).

### 3.2 Phylotyping and MLST

WGS data of the 58 *E. coli* isolates collected from humans, livestock and wildlife enabled the generation of genome assemblies, resulting in a mean genome coverage of 178.98 (SD = 53.58). The Clermont phylotyping scheme identified 20 isolates belonging to group B1 (34.5%), 16 to group A (27.6%), 12 to group D (20.7%), 3 to group U (5.2%), 2 each to group B2 and E (3.4%), and 1 each to group C and F (1.7%). One isolate could not be classified. Phylotypes A, B1 and D were found in wildlife samples. Furthermore, a remarkable range of ESC-resistant *E. coli* lineages carrying different AMR genes were identified, comprising 37 different sequence types (STs) (Supplementary Table S3). ST6636 was the most common (*n* = 5, 9.4%), followed by ST2178 (*n* = 4, 7.5%), ST2852 (*n* = 3, 5.7%), ST10 (*n* = 2, 3.8%), ST224 (n = 2, 3.8%), ST295 (n = 2, 3.8%), ST720 (n = 2, 3.8%), ST1312 (n = 2, 3.8%), ST2705 (n = 2, 3.8%) and ST9138 (n = 2, 3.8%). ST6636 was detected in a Ugandan kob, while ST10, ST224 and ST2705 were found in baboon samples. ST131 was identified in one human sample. Four *E. coli* isolates did not have ST assignments as they did not meet the quality control standards of Enterobase.

Among the *Klebsiella* spp. isolates, the three *K. pneumoniae* strains were identified as ST16, ST1411, and ST5405, whereas the two *K. quasipneumoniae* corresponded to ST414 and ST841.

### 3.3 Phylogenomic reconstructions

**Fig. 2:**
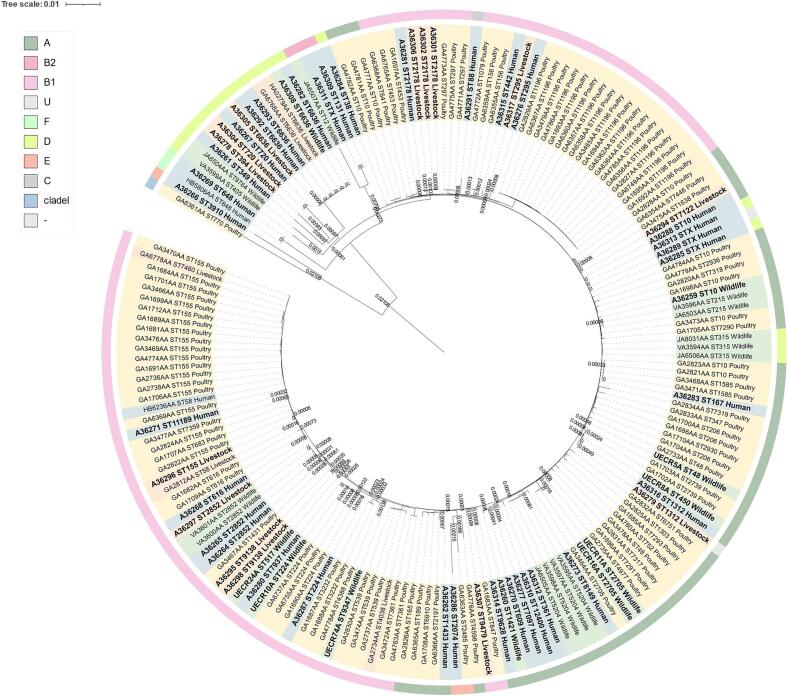
Phylogenetic reconstruction of *E. coli* genomic sequences isolated from humans, livestock and wildlife sampled in Western Uganda, and *E. coli* genomic assemblies available at Enterobase from human, livestock and wildlife origin (https://enterobase.warwick.ac.uk/, last access 23/06/2024).

The *E. coli* phylogenomic tree (Fig. 2) included a total of 182 assemblies (58 from this study and 124 from public databases, all with Ugandan origin). The phylogenetic analysis revealed the presence of two clades, the main one containing all sequences except for two. The main clade clustered the *E. coli* isolates by ST and, to a lesser extent, by phylotype. Some clusters contained closely related isolates from different sources, according to the phylogenetic distance obtained from the core genome. The chimpanzee isolate UECR8A shared homology with isolates of human (A36316, 17 different SNPs) and goat (A36279, 16 different SNPs) origin. While both chimpanzee and goat samples were from BCFR, the human isolate belonged to an individual sampled in MGNP. Additionally, a livestock isolate from QENP (A36297) was closely related to two isolates of wildlife origin from central Uganda (VA3601AA and VA3600AA, 0 different SNPs). Genetically similar ST6636 isolates were observed between a Ugandan kob (A36308) from QENP, and a human and cattle isolates from MGNP (0 or 1 different SNPs). Furthermore, homology was also observed among a human and cattle isolates (A36267 and A36304, 0 different SNPs), harbouring CTX-M-15 predicted to be located in the same mobile genetic element.

**Fig. 3:**
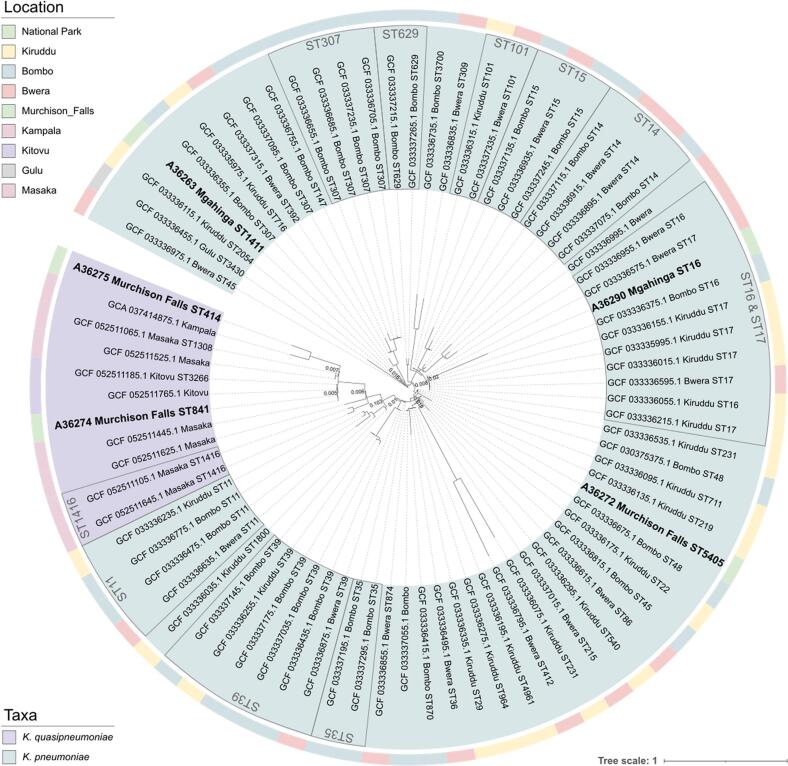
Phylogenetic reconstruction of *Klebsiella* spp. genomic sequences isolated from humans in Western Uganda, and *K. pneumoniae* and *K. quasipneumoniae* assemblies available at NCBI from Uganda (last access 23/09/2025).

Phylogenetic analysis of *Klebsiella* spp. isolates included a total of 81 assemblies, 70 *K. pneumoniae* isolates (3 of this study), and 11 *K. quasipneumoniae* isolates (2 of this study) (Fig. 3). The phylogenetic tree revealed a clear separation between the two species, with further clustering within each species based on their STs. A broad diversity of STs was observed across the collection, with the most prevalent STs being ST17, ST39, and ST307 (*n* = 6 each, 8.6%), followed by the ST11, ST14, and ST16 (*n* = 4 each, 5.7%) for *K. pneumoniae*, and ST1416 (*n* = 2, 18.2%) for *K. quasipneuamoniae*. The isolates from our study were geographically distant from those clustering nearby in the phylogenetic tree. However, the human isolate A36290 from our study showed notable genomic similarity to an ST16 isolate from a patient at the General Military Hospital of Bombo, differing by only 29 SNPs and suggesting restricted geographic spread of this lineage.

## 4 Discussion

Antimicrobial resistance poses a significant challenge in Uganda, where limited resources and complex interactions between humans, wildlife, and livestock exacerbate the spread of resistant bacteria. In regions with limited healthcare infrastructure and diverse ecological landscapes, AMR threatens public health efforts. This study addresses these challenges by investigating the occurrence, phenotypic and genotypic resistance, and potential origins of ESC Enterobacterales, including *E. coli* and *Klebsiella* spp., as indicators of AMR in humans, livestock and wildlife across Western Uganda.

The antibiotic resistance profiles revealed a high percentage of isolates from humans and animals exhibiting resistance to commonly used antibiotics, such as ampicillin, sulfamethoxazole, and tetracyclines [43]. However, isolates collected from water, representing environmental samples, were pan-susceptible. In all cases, proportions of resistance were higher in humans, followed by livestock and wildlife. Levels of phenotypic resistance in *E. coli* isolated from humans were similar to other studies performed in Uganda [44]. While resistance frequency in cattle was lower than previously observed [44], [45], resistance frequency in *E. coli* of goat and sheep origin and wildlife isolates, was higher [45]. A high frequency of resistance to third-generation cephalosporins, which are widely prescribed in Ugandan hospitals [46], was observed in both humans and animals, including different wildlife species. Furthermore, all ESBL-producing isolates were MDR. The presence of these resistant isolates was significantly higher in areas with higher interaction between species, where rural interfaces could facilitate the transmission of AMR through direct contact among species, consumption of contaminated meat through game and home slaughter, and shared contaminated environments [20], [47]. Although no significant differences were observed in wildlife from areas with varying levels of interaction, BCFR exhibited the highest proportion of ESC-resistant *E. coli* isolates, predominantly found in non-human primates. This is concerning not only because of the close proximity of these species to human settlements, but also because of social interactions among non-human primates. Besides facilitating pathogen exposure, as previously reported in these species, these interactions can also promote the transmission and maintenance of AMR within the same group [48], [49].

The characterization of ESC genes in Uganda is poorly documented, particularly in wild animals. In this study, we report the presence of MDR ESBL-producing *E. coli* harbouring different CTX-M genes in humans, livestock and wildlife, with CTX-M-15 being the most common in all species. Resistance genes belonging to the CTX-M-1 group have been previously detected in humans in Uganda [50], while CTX-M-15, CTX-M-27 and OXA-1 have been detected in cattle [51], [52]. The acquisition of AMR in humans and livestock could be driven primarily by the selective pressure from the widespread and inadequate use of antibiotics in Uganda, which enables the rapid spread of resistance genes among bacterial populations [51]. Those resistance genes could subsequently be excreted and dispersed into the environment through agricultural and human sources, facilitating the dissemination of AMR to wildlife [53]. The significance of AMR in Uganda is underscored by the close interactions between humans and animals, particularly in regions adjacent to national parks. The absence of effective barriers, along with the degradation of natural habitats due to agricultural expansion, facilitates these interactions, thereby enhancing the risk of pathogens and AMR transmission [45], [54]. Despite CTX-M-15 being previously detected in wildlife, specifically in captive and wild chimpanzees from Uganda [52], this study constitutes the first report of MDR *E. coli* in an antelope (Ugandan kob) producing CTX-M-15. CTX-M-15 is widely disseminated worldwide, mostly related to healthcare setting infections [55]. However, this gene has also been detected in wild birds and ungulates in different European and African countries, leading to the hypothesis that their presence in wildlife species could be attributed to anthropogenic activities [11], [12], [56]. In addition, OXA-1 was detected in humans from different regions of Uganda and in a chimpanzee from BCFR, where encounters with human settlements are common as some chimpanzees leave the forest to obtain food from croplands [57]. *E. coli* and *Klebsiella* spp. isolates also carried multiple AMR genes for other antibiotic families, such as aminoglycosides, trimethoprim and sulphonamides, with all being MDR. In addition, isolates from human, goat and a chimpanzee origins exhibited plasmid-mediated quinolone resistance (PMQR) determinants, which reduce the susceptibility of bacteria to quinolones, facilitating the selection of quinolone-resistant mutants and promoting therapeutic failure across Enterobacterales species [58]. The studied areas represent intricate ecosystems that facilitate the easy spread of resistant pathogens, posing significant challenges to public, animal and environmental health. Despite ongoing national efforts to strengthen antimicrobial stewardship and infection control [59], addressing AMR in these rural settings requires comprehensive strategies that integrate environmental, agricultural, and healthcare practices to effectively mitigate the spread of resistance.

Surprisingly, 38 different ST lineages were detected, with only six aligning with the predominant extraintestinal pathogenic *E. coli* (ExPEC) lineages commonly reported in humans globally: ST10, ST38, ST88, ST131, ST167 and ST648 [60]. Each one of these lineages was represented in human isolates sequenced herein, while ST10 was also detected in a baboon isolate. Despite ST131 being a MDR lineage distributed worldwide and found in a wide host range [61], it was only detected in a human isolate from the northern sector of QENP, in Western Uganda. Furthermore, most beta-lactam resistance genes were predicted to be located in plasmids. In fact, the high diversity of *E. coli* lineages detected suggests that beta-lactam resistance is spreading through mobile genetic elements. This is of great concern as plasmid transfer allows for a more rapid dissemination of resistance genes between diverse lineages and taxa [62], [63], posing a significant challenge for the control and treatment of bacterial infections in Uganda. Additionally, plasmids can carry multiple resistance genes, enabling the propagation of MDR isolates [64]. In the present study, some resistance genes detected in samples from humans, livestock and wildlife, which are predicted to be located on plasmids, exhibited resistance not only to beta-lactams but also to quinolones, aminoglycosides, and sulphonamides, posing a risk of MDR spread. Reports of *E. coli* ST6636 are rare. However, isolates A36282, A36292 and A36283 (human), A36305 (cattle) and A36308 (Ugandan kob) exhibited 100% core genome homology with Ugandan *E. coli* strains from dairy cattle (Bioprojects PRJNA293225) on Enterobase [65]. Furthermore, almost all isolates carried the CTX-M-15-encoding gene. Similarly, the core genome of a livestock isolate of lineage ST2852 B1 CTX-M-15 was identical to an isolate of chimpanzee origin from Ngamba Island Sanctuary, located in lake Victoria, which also carried the CTX-M-15-encoding gene [52]. These findings not only indicate that resistance genes can spread across different species but also underscore the significance of transmission pathways, including the influence of anthropogenic pressures in wildlife and the role of natural environmental contamination in the dissemination of antimicrobial resistance genes.

Despite their low frequency of detection, the identification of *E. coli* ExPEC lineages in human isolates underscores a critical concern. These strains contribute substantially to the global burden of infectious diseases and act as a reservoir for the development and dissemination of AMR [60]. Particularly in LMICs, the impact of such pathogens is exacerbated by limited healthcare resources and higher rates of infectious diseases, posing the risk of them becoming untreatable.

In our study, MDR *Klebsiella* spp. was detected in five human samples, three *K. pneumoniae* and two *K. quasipneumoniae*, and five different ST lineages. ST16, which is considered an important emergent lineage carrying determinants of carbapenem-resistance and causing hospital outbreaks worldwide [66], [67], [68], was identified in one *K. pneumoniae* isolate harbouring CTX-M-15 and OXA-1 encoding genes. In addition, other three isolates (two *K. quasipneumoniae* and one *K. pneumoniae*) carried the CTX-M-15-encoding gene. Point mutations that could alter the outer membrane porin OmpK36 were detected in all isolates, which play a critical role in mediating *Klebsiella* spp. resistance to carbapenems [69]. *K. pneumoniae* is considered an important reservoir of diverse AMR genes worldwide, most of which are plasmid-borne and transmitted via conjugation, leading to a wider ecological distribution than other opportunistic bacteria and the emergence of extremely drug resistant strains, as observed in our three *K. pneumoniae* isolates compared to the *K. quasipneumoniae* isolates [70], [71]. While some bacteria originate from human and animal sources, *K. pneumoniae* is ubiquitous in nature and can originate from various natural sources [72]. In our phylogenetic analysis, all isolates were of human origin, although with high variability in ST lineages. However, since our isolates were obtained from apparently healthy individuals, and both our study and the publicly available assemblies included samples from multiple geographic locations within Uganda, this suggests that *Klebsiella* spp. are widespread across the country and have potential for further dissemination. Consequently, this pathogen plays a crucial role in AMR spread from environmental microbes to clinically critical pathogens. In fact, *K. pneumoniae* has been classified as an ESKAPE organism, which includes *Enterococcus faecium, Staphylococcus aureus, K. pneumoniae, Acinetobacter baumannii, Pseudomonas aeruginosa,* and *Enterobacter* species. These pathogens are the leading causes of nosocomial infections worldwide and are predominantly MDR [73]. Within a One Health framework, all these microorganisms have the potential to disseminate AMR genes across the different One Health domains, highlighting the broader need of monitoring ESKAPE pathogens across human, animal, and environmental interfaces [74]. Despite the alarming increase in MDR *K. pneumoniae* isolates in humans reported over the last decades, their prevalence in domestic and wild animals, as well as in the environment, has not been significantly investigated. Therefore, further research is imperative to understand its ecological distribution and to develop effective measures to control their spread.

## 5 Conclusions

In conclusion, the wide occurrence of ESC-resistant bacteria in community settings and wildlife highlights their capacity to spread between anthropogenic and natural ecosystems, creating hotspots that facilitate the dissemination and evolution of AMR genes in Western Uganda. The detection of resistance genes in wildlife not only suggests that these animals may act as reservoirs but also highlights the potential risks to environmental health. Proximity to human settlements and livestock farming contributes significantly to this issue by increasing the selective pressure for AMR and facilitating its spread, underscoring the need to adopt an expanded One Health approach to fully understand AMR transmission dynamics in rural areas from Eastern Africa.

## Data sharing statement

All genome sequences of the 58 ESC-resistant *E. coli* isolates of humans, livestock and wildlife from Western Uganda have been deposited in the Enterobase (Accession numbers in Supplementary Table S3).

## CRediT authorship contribution statement

**Judith Guitart-Matas:** Writing – review & editing, Writing – original draft, Methodology, Investigation, Formal analysis, Data curation, Conceptualization. **Andrea Dias-Alves:** Writing – review & editing, Writing – original draft, Methodology, Investigation, Formal analysis, Data curation, Conceptualization. **Ignasi Marco:** Writing – review & editing, Supervision, Resources, Project administration, Data curation, Conceptualization. **Laura Carrera-Faja:** Writing – review & editing, Methodology, Investigation, Formal analysis. **Jesús Muro:** Writing – review & editing, Resources, Methodology. **Josephine Tushabe:** Writing – review & editing, Resources, Methodology. **Edrine Kayaga:** Writing – review & editing, Methodology, Investigation. **Dickson Ndoboli:** Writing – review & editing, Methodology, Investigation. **Fred Babweteera:** Writing – review & editing, Methodology. **Carol Asiimwe:** Writing – review & editing, Methodology. **Oscar Cabezón:** Writing – review & editing, Validation, Supervision, Resources, Methodology, Investigation, Formal analysis, Data curation, Conceptualization. **Lourdes Migura-Garcia:** Writing – review & editing, Writing – original draft, Validation, Supervision, Resources, Methodology, Investigation, Formal analysis, Data curation, Conceptualization.

## Funding sources

This study was funded by “Fundació Autònoma Solidària (FAS)” from Universitat Autònoma de Barcelona (Spain) within project FSXXXIV-05. Andrea Dias-Alves acknowledges the Government of Andorra from a predoctoral grant (ATC020-AND-2020/2021 and ATC020-AND-2021/2022).

## Declaration of competing interest

The authors declare no conflict of interest.

## Data Availability

Data will be made available on request.
